# Genetic, Biochemical and Clinical Insights into Primary Congenital Glaucoma

**DOI:** 10.5005/jp-journals-10008-1140

**Published:** 2013-05-09

**Authors:** Muneeb Faiq, Reetika Sharma, Rima Dada, Kuldeep Mohanty, Daman Saluja, Tanuj Dada

**Affiliations:** Pursuing Doctorate, Dr Rajendra Prasad Centre for Ophthalmic Sciences, All India Institute of Medical Sciences, New Delhi, India; Resident, Dr Rajendra Prasad Centre for Ophthalmic Sciences, All India Institute of Medical Sciences, New Delhi, India; Additional Professor, Department of Anatomy, Laboratory for Molecular Reproduction and Genetics, All India Institute of Medical Sciences, New Delhi, India; Pursuing Doctorate, Dr Rajendra Prasad Centre for Ophthalmic Sciences, All India Institute of Medical Sciences, New Delhi, India; Professor, Medical Biotechnology Laboratory, Dr BR Ambedkar Centre for Biomedical Research, University of Delhi, New Delhi, India; Additional Professor, Dr Rajendra Prasad Centre for Ophthalmic Sciences, All India Institute of Medical Sciences, New Delhi, India

**Keywords:** Glaucoma, Primary congenital glaucoma, Genetics, CYP1B1, Myocilin.

## Abstract

Glaucoma is an irreversible form of optic neuropathy in which the optic nerve suffers damage in a characteristic manner with optic nerve cupping and retinal ganglion cell death. Primary congenital glaucoma (PCG) is an idiopathic irreversible childhood blinding disorder which manifests at birth or within the first year of life. PCG presents with a classical triad of symptoms (*viz* epiphora, photophobia and blepharospasm) though there are many additional symptoms, including large eye ball and hazy cornea. The only anatomical anomaly found in PCG is trabecular meshwork (TM) dysgenesis. PCG is an inheritable disease with established genetic etiology. It transmits through autosomal recessive mode. A number of cases are sporadic also. Mutations in many genes have been found to be causative in PCG and many are yet to be found. Mutations in cytochrome P4501B1 (CYP1B1) gene have been found to be the predominant cause of PCG. Other genes that have been implicated in PCG etiology are myocilin, Forkhead-related transcription factor C1 (FOXC1) and latent transforming growth factor beta-binding protein 2 (LTBP2). Mutations in these genes have been reported from many parts of the world. In addition to this, mitochondrial genome mutations are also thought to be involved in its pathogenesis. There appears to be some mechanism involving more than one genetic factor. In this review, we will discuss the various clinical, biochemical and genetic aspects of PCG. We emphasize that etiology of PCG does not lie in a single gene or genetic factor. Research needs to be oriented into a direction where gene-gene interactions, ocular embryology, ophthalmic metabolism and systemic oxidative status need to be studied in order to understand this disorder. We also accentuate the need for ophthalmic genetic facilities in all ophthalmology setups.

**How to cite this article:** Faiq M, Sharma R, Dada R, Mohanty K, Saluja D, Dada T. Genetic, Biochemical and Clinical Insights into Primary Congenital Glaucoma. J Current Glau Prac 2013;7(2):66-84.

## INTRODUCTION

**Fig. 1:**
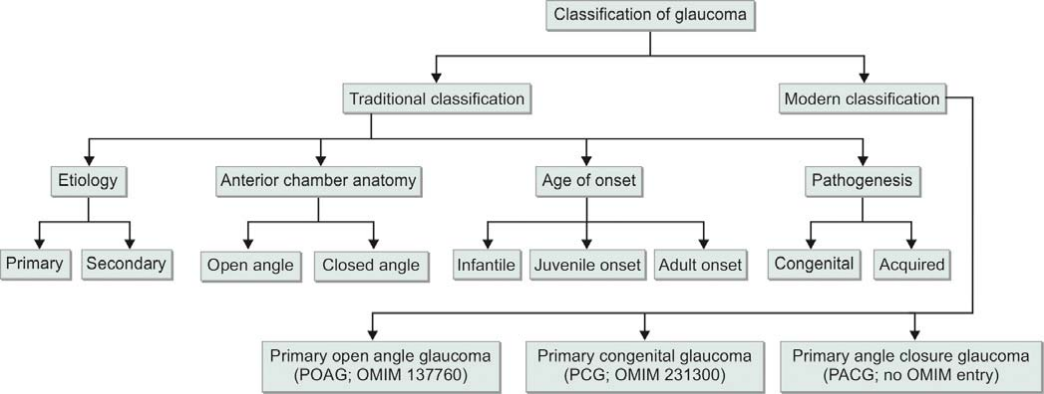
General classification of glaucoma

Glaucoma (pronunciation: glaw-ko'me),^1^ derived from Greek *glaukos* (meaning bluish-green gleam)^2^ is a term referring to a collection of related disorders with complex optic nerve atrophy^3^ and characteristic loss of larger retinal ganglion cells (RGC)^4,5^ leading to a consequent carbon copy pattern of loss of visual field and vision.^6^ The bluish green gleam was initially observed during dilated pupil eye examinations.^7^ Glaucoma has been nicknamed as ‘the sneak thief of sight' and is characterized by those ocular conditions in which the intraocular pressure (IOP) is too high for the normal functioning of the optic nerve head. Previously, it was thought that glaucoma is the disease of the lens and hence the term was used for cataract.^7^ Later, the term buphthalmos found its way into literature which meant the enlargement of the ocular globe that resembled the eye of an ox (Greek *bous* means ox).^8^ Increase in the aqueous humor accumulation in the anterior chamber was recognized as an important clinical feature of glaucoma.^8^ It was then Adolf Weber who in 1856 elaborated the glaucomatous cupping of the optic disk.^8^ Glaucoma is the second largest cause of blindness in the world affecting an estimated population of 60 million.^9,10^ It has been estimated that there are 1.5 to 2 million blind children in the world and a majority of them live in developing countries.^11^ With regards to Indian scenario, Balasubramanian et al estimated that 1.8% of the Indian population is blind and 0.15% suffers from glaucoma.^12^ Once glaucomatous blindness is precipitated, there is no known treatment to refurbish vision. As a matter of fact, blindness from glaucoma is preventable in almost all cases. What is required for this prevention is early detection and prompt treatment. Early diagnosis with genetic counseling and proper molecular workout is likely to bring down the prevalence of glaucomatous blindness.^13^ If obstetricians make it a routine to observe the eyes of the neonate; early diagnosis on childhood glaucomas can be enhanced and consequent blindness hampered. There have been a few attempts to classify glaucomas but no classification system is absolute. However, glaucoma is traditionally classified on the basis of etiology (primary and secondary), anatomy of anterior chamber (open angle and closed angle), time of onset (infantile, juvenile and adult) and pathogenesis (congenital and acquired). Nevertheless, modern classification system classifies this disorder into three major groups *viz* (a) primary open angle glaucoma (POAG; OMIM 137760), (b) primary congenital glaucoma (PCG; OMIM 231300) and (c) primary angle closure glaucoma (PACG; no OMIM entry). Figure 1 depicts the general outline of the classification of glaucoma. The etiology of a majority of glaucoma cases is unknown^14^ but many factors like increase in IOP, obstruction to aqueous humor drainage, development anomalies of anterior chamber^15,16^ and genetic susceptibility have been implicated. One of the prime focuses in our laboratory is to understand, appreciate and get an insight into the genetic etiopathology of the disease and its correlation with the so developed phenotype. We endeavor to correlate gene mutations with severity of the phenotype, age of onset, response to medical (IOP lowering drugs like P-blockers) and surgical therapy (trabeculectomy/trabeculotomy) giving considerable insight into the prognosis and management of glaucoma. Genotype phenotype correlations are likely to help clinicians manage the disease better and the genetic counselor to help inflicted families with the disease management.^13^

## PRIMARY CONGENITAL GLAUCOMA

Primary congenital glaucoma (PCG; OMIM 231300; provided in the public domain by National center for Biotechnology Information, Bethesda, MD) is an autosomal recessively inherited severe form of glaucoma resulting from obstruction in the aqueous humor drainage due to congenital developmental anomalies in anterior chamber angle/angle structures.^17-19^ It accounts for 22.2% of all pediatric glaucoma cases. Congenital buphthalmos has early history having been recognized since the time of Hippocrates (460-377 BC), Celsus (1st century CE) and Galen (130-201 CE). Buphthalmos is a result of the distensibility of the neonatal eye ball. This distensibility in the neonatal eye ball is because of the high content of elastic fibers in sclera. The mechanism of buphthalmos includes global delocalized stretching of the sclera, optic nerve and the related structures due to increased IOP.^20,21^ The normal range for corneal diameter of a neonate is 10 to 10.5 mm which, owing to growth, increases by 0.5 to 1.0 mm in first year of life.^22^ Any increase in corneal diameter (>12 mm) in the first year of life is suggestive of PCG.^21^ Additionally, the optic nerve cupping being one of the hallmarks of glaucoma; in case of PCG, the optic nerve changes are not similar to that of glaucomatous adults. Optic nerve cupping may progress fast and early in newborns and toddlers^23,24^ and can be reversible if normal IOP is restored well in time.^25^ On the other hand, glaucomatous optic nerve head damage is irreversible in adults.^24^

**Figs 2A to D:**
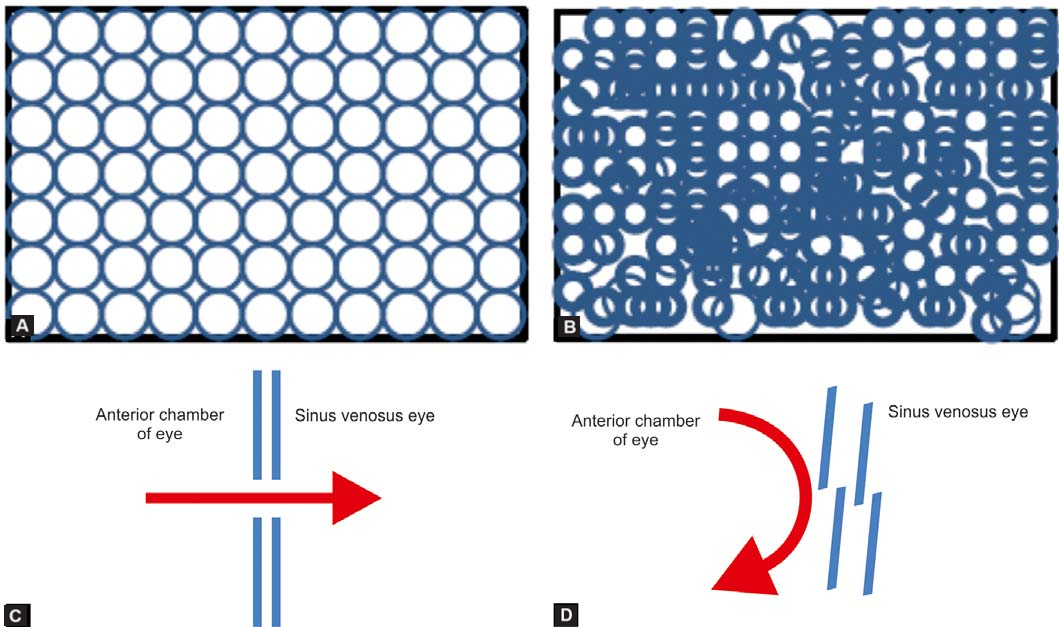
Schematic representation of the trabecular meshwork dysgenesis and mechanism of IOP elevation: (A) Normal trabecular meshwork, (B) glaucomatous trabecular meshwork, (C) smooth unobstructed flow of aqueous humor across the trabecular meshwork, (D) obstruction in the aqueous flow and consequent build up of IOP

PCG has its onset at as early as birth or manifests within first 3 years of life.^26^ It presents with a classical triad of symptoms *viz* epiphora (excessive tearing), photophobia (hypersensitivity to light) and blepharospasm (inflammation of the eyelids).^21^ Any combination of these symptoms are indicative (if not conclusive) of glaucoma. These symptoms are caused by the irritation of cornea leading to corneal epithelial edema and haze (clouding). Enlargement of eye (buphthalmos) is a result of increased IOP. The other findings include: (i) iris covering a variable portion of ciliary body and trabecular meshwork (TM),^19,27^ (ii) thickened trabecular beams in trabeculum^28^ as schematically depicted in Figure 2, (iii) juxtacanalicular meshwork with very small number of pores,^29,30^ (iv) absence of sinus venosus eye (i.e. Schlemm's canal)^17,31^ and (v) breaks in Descemet's membrane (Haab's striae).^7^ A collection of the symptoms of PCG are depicted in Table 1. The term PCG has been restricted to the cases where only anatomical defect observed is isolated trabecular dysgenesis.^32^ This is also referred to as isolated congenital glaucoma.^33^ A characteristic cloudiness or foggy manifestation of the cornea can be observed in early stages which appear before any breaks in the Descemet's membrane are visible. PCG comprises 4.2% of all childhood blindness being bilateral in 80% of cases. PCG cases presented within first year of life form more than 80% out of which 25% are diagnosed in the neonatal period and about 60% within first 6 months of life. Being the most common pediatric glaucoma, PCG comprises more than 55% of primary pediatric glaucomas with expression and penetrance varying from 40 to 100%.^34^

**Table 1:** **Table 1:** Symptoms of PCG

	*Classical triad of symptoms*			
	*Symptom*		*Description*	
	1. Epiphora		Excessive tearing	
	2. Photophobia		Hypersensitivity to light	
	3. Blepharospasm		Inflammation of eyelids	
	*Additional signs and symptoms*			
	1. Enlargement of the eyeball (buphthalmos)			
	2. Iris covering a variable portion of ciliary body and trabecular meshwork			
	3. Juxtacanalicular with less number of pores			
	4. Absence of Schlemm's canal (sinus venosus eye)			
	5. Breaks in Descemet's membrane (Haab's striae)			

### Genetics of PCG

The first report on genetic predisposition to glaucomas was published in 1842 when Benedict observed two sisters suffering from glaucoma.^35^ Afterward, many reports came to the limelight suggesting a definite role of genetic factors.^36-38^ PCG is inherited in an autosomal (of chromosomes other than sex chromosomes) recessive mode of transmission. Association of congenital glaucoma with chromosomal abnormalities of at least 17 different autosomes has also been reported in literature.^39^ Congenital glaucoma being associated with chromosomal aberrations has been well documented in many reports.^40-42^ To quote examples, congenital glaucoma with 22p+ variant chromosome has been reported in a study of Indian population.^42^ Another study has observed various chromosomal anomalies, such as trisomy 8q22-qter/monosomy 9p23-qter in Australian population.^43^ Additionally, Mitchell et al^44^ has observed 4q deletion in Axenfeld-Rieger's syndrome (ARS). We have also reported 4q deletion in a case of ARS.^45^ Moore et al reported 11p deletion in aniridia (absence of iris).^46^

The genetic basis of PCG remains idiopathic but 13 chromosomal loci (GLC1A to GLC1N) for the POAG and three chromosomal loci (GLC3A to GLC3C) for PCG have been mapped. Out of these 13, only 4 *viz* GLC1A (myocilin gene) and GLC1E (optineurin gene) for POAG and GLC3A (CYP1B1 gene)^47-49^ and GLC3C (LTBP2 gene) for PCG have been characterized. Independent studies by many researchers during the past few years have lead to the mapping of three distinct ‘GLC' loci for PCG. ‘GLC' is the nomenclature of the Human Genome Organization (HGO) for glaucoma. The numerals ‘1', ‘2' and ‘3' following ‘GLC' refer to juvenile open angle glaucoma (JOAG), angle closure glaucoma and congenital glaucoma respectively. The letters ‘A', ‘B' and ‘C' following the numerals indicate the chronology (date-wise) in which the genes were mapped. Table 2 summarizes various aspects of currently known‘GLC' loci with their respective mode of inheritance and penetrance. PCG is thought to be transmitted as an autosomal recessive trait.^50^ This is because lower than expected number of cases have been observed and genetic heterogeneity has been postulated with support from linkage studies.^51^ The unequal sex distribution of PCG, however, points to some additional unknown factors. Majority of PCG cases are sporadic but 10 to 40% are familial and associated with consanguinity.^34^ The expression and penetrance of this disorder varies from 40 to 100%. Up until now, three genetic loci have been mapped for PCG.^52,53^ PCG presents a differentially variable geographical distribution with lower occurrence in western countries and high prevalence in the middle-east and still higher prevalence in consanguineous societies like Slovakian gypsies^54^ and Saudi Arabians. The manifestations of PCG show a particular concordance in monozygotic twins and discordance in dizygotic twins.^50^ Prevalence of PCG ranges from one in 10,000 in Europe^55^ and one in 3,300 in Andhra Pradesh (Southern India)^56^ to one in 2,500 in middle-east (Saudi Arabia) and one in 1,250 in Roms (Gypsy population of Slovakia).^55,57-59^ Though transmitted through autosomal recessive mode as suggested by many reports,^52,60-62^ this mode of transmission of PCG has recently been questioned^63^ because of differential prevalence in different sexes.^64^ Prevalence among males accounts for 65% of total number of cases.^65^ In an early study from Japan females were, conversely, reported to be having higher prevalence.^66^ On the other hand, males and females were reported to be equally affected in Europe and USA.^67^ In a study, on 616 cases of congenital glaucoma where patients were divided into two groups (one with sporadic cases and other with familial), it was observed that, in the first group there were 70% of males and the second group comprised of 58% males. PCG has been shown to display a definite pattern of inheritance in 30 to 40% cases^67^ but some investigators have reported familial incidence of only 11 to 14%.^68^ There are, nonetheless, so many facts and observations that indicate the autosomal mode of transmission of PCG with a penetrance of 40 to 100%. The autosomal recessive mode of PCG transmission has been demonstrated in rabbits^69-71^ and dogs.^72^ Intermediate inheritance has also been reported in rabbits.^73^ One more observation supporting the autosomal recessive means of inheritance is that, in PCG, the incidence of consanguinity of the parents overshoots 8%.^74^ There are so many studies that support this observation.^68,75-78^ Moreover, in most of the PCG cases, two or more siblings are affected with normal parents and descendents.^68,74,76^ A classical report by Gianferrari and his coinvestigators document two most characteristic pedigrees in which the first consisted of 11 cases of buphthalmos and two consanguineous marriages with three affected children out of four and two out of 12.^75^ And, in case of the other pedigree, there were five cases of congenital glaucoma and two consanguineous marriages with two affected children out of four and one out of six.^75^ A peculiar finding in these pedigrees supporting autosomal recessive mode of inheritance is that the parents were phenotypically normal in all these cases. PCG is mostly sporadic with variable penetrance.^7,33,68,75,79-83^

**Table 2:** **Table 2:** Mapping and clinical features of PCG

*Nomenclature*		*Chromosomal location*		*DNA marker region*		*Gene identified*		*Mode of inheritance*		*Penetrance*	
GLC3A		2p21-22		D252186 and C251346		CYP1B1		Autosomal recessive		Severe	
GLC3B		1p36		D151597 and D151176				Autosomal recessive		Severe	
GLC3C		14q24.3		D14553		LTBP2		Autosomal recessive		Severe	

These observations are thought to exist because of genetic heterogeneity.^51,84^ The genetic heterogeneity studies for PCG started in early 1990s and the first locus for PCG (GLC3A) was mapped to chromosomal location 2p21 in 1995 by Sarfarazi et al.^60^ The investigators described the involvement of this locus in 11 of the 17 Turkish families studied. They performed haplotype analysis and homozygosity mapping which led to the mapping of the diseased gene within 2.5 cM interval flanked by two DNA marker regions *viz* D252186 and C251346. Since this was the first chromosomal locus identified to be associated with PCG, it was designated ‘GLC3A' (GLC: glaucoma, 3: congenital, A: first locus identified) as per the nomenclature for HGO/Genome Database. Later studies done on Slovakian and Saudi Arabian population confirmed this finding.^61,62^ Since only 11 of the 17 Turkish families were found to be having involvement of 2p21 region in their disease phenotype, it was therefore, logical to think that at least one more locus existed to account for the remaining six families. On this premise, the investigators performed haplotype analysis of these six families and successfully mapped the disease locus of four of these families to the chromosomal location 1p36 within in gene interval of 3 cM. This locus was found to be flanked by two sets (six in total) of DNA marker regions *viz* D151597/D15485/D15228 and D151176/D15508/D15407. There are many tumor suppressor genes in this region.^85^ Being the second locus to be mapped, it was designated ‘GLC3B'.^52^ A very large number of genes have been found to be located in 1p36 region but none has been postulated as a causative factor for PCG. This region is very prone to chromosomal aberrations resulting in many malignant diseases. Also, the neighboring regions of 1p36 quite often participate in recombination events.^86^ Since the identification of genetic locus for two of the 17 families still remained, it meant that at least one more genetic locus remains to be mapped. The multigeneration pedigree analysis of one of those remaining two families revealed a third PCG locus with chromosomal location 14q24.3 within a region 2.9-cM narrow.^53^ Being the third in the chronology of mapping, this locus was designated ‘GLC3C'. Using the genome-wide scanning approach, the investigators were able to identify the DNA markers region D14553. Using additional DNA markers in haplotype analysis, it was found that all PCG patients shared DNA marker regions of homozygosity including D14542/ D145983/D1451020 and D14574.

### Polygenic Inheritance of PCG

Before going on to discuss the genes involved and implicated in PCG, it forms a good rationale to add a note about its polygenic inheritance. PCG is not a unigenic etiological outcome but rather multifactorial genetic phenotype. Many genes (CYP1B1, MYOC, FOXC1, LTBP2, etc.) have been found to be involved in its etiology but there remains a great spectrum to be identified. Mutations in CYP1B1 gene account for a very little percentage of total number of PCG cases, it is logical for that reason to speculate about the role of other genes in PCG development and pathogenesis. There are a number of studies which suggest that the bequest of PCG is polygenic (involving more than one gene as an etiological factor). These genes may have functional interactions among themselves and may, for that reason, act in synergy with each other leading to the development of anterior chamber ocular structures. Any violation of the harmony anywhere in the functional muddle of these genes may give rise to anomalies in the anterior chamber anatomy and, therefore, to PCG. Polygenic inheritance can be explained via many reasons, such as variable penetrance observed in these cases and varied prevalence with respect to sex in different population. There are, however, some studies which suggest that there are no gender-wise differences in prevalence of PCG.^82^ As we will discuss in this review that many genes have been implicated in etiology of PCG, the polygenic etiology of this disorder is well confirmed. Functional interaction between CYP1B1 gene and MYOC gene further lends support to this.

### Cytochrome P450, Family 1, Subfamily B, Polypeptide 1 (CYP1B1) Gene and PCG

The cytochrome P450 superfamily consists of as many as 70 gene families spanning across bacteria, yeasts, insects and vertebrates as well as plant kingdom.^87^ Fourteen mammalian families of P450 have been observed till date, out of which, five have been reported to have many subfamilies. Formerly, it was thought that cytochrome-P4501B1 family is composed of two members *viz* CYP1A1 and CYP1A2.^87^ The genes of this family are ubiquitous in mammals and have comparable catabolic activities toward a great number of xenobiotics. CYP1B1 is the third member of this family which has been recently characterized. CYP1B1 gene (GenBank accession no. U56438), therefore, forms a member of the superfamily of drug metabolizing enzymes cytochrome–P450 (CYP450). The CYP450 family is inclusive of 58 functional genes in humans and 102 in mice.^88^ Initially, this gene was recognized as a dioxin-responsive cDNA clone. The activities of this gene against various xenobiotics and metabolic approaches toward many procarcinogens have been thoroughly explored.^89^ In one of the approaches, TCDD (2,3,7,8-tetrachlorodibenzo-p-dioxin) responsive cDNA was isolated from a human keratinocyte cell line and established as a new cytochrome P450 superfamily member.^89^ The translation product of this gene designated as cytochrome P4501B1 (CYP1B1) has also been cloned and characterized from mouse^90^ and rat.^91^ It is interesting to note that the size of rodent and human CYP1B1 mRNA is almost equal (5.2 kB in mice and 5.1 kB in humans). Furthermore, both rodent and human CYP1B1 code for a protein 543 amino acids long. CYP1B1 is constitutively expressed in adrenal glands, ovaries, testes and more than 15 other tissues of the body. Its expression can be upregulated by aromatic hydrocarbons, adreno-corticotropin and peptide hormones.^89,92^ This protein plays a pivotal role in metabolism of drugs and a wide range of xenobiotics.^92^ The tissue distribution pattern of this protein suggests its role in metabolism of steroid hormones^92,93^ at least indirectly. CYP1B1 gene was isolated and mapped to chromosomal location 2p21-22 by Tang et al.^94^

**Fig. 3:**
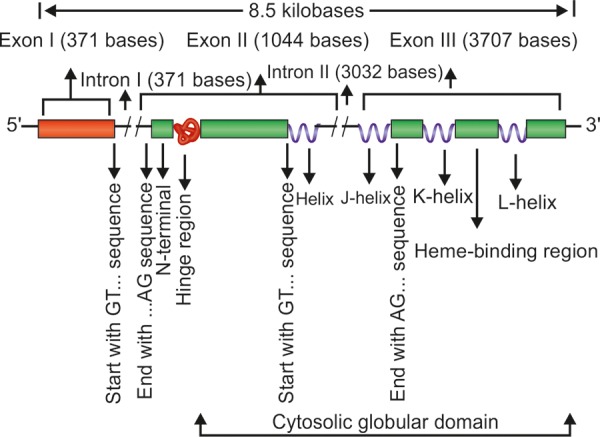
Schematic representation of CYP1B1 gene

In humans, the CYP1B1 gene consists of three exons (with open reading frame starting from exon II) and two introns and is 8.5 kB long. These three exons are 371, 1,044 and 3,707 base pairs in length respectively. Conversely, the two introns are 390 and 3,032 base pairs long. Both the introns commence with the sequence GT… and end with the sequence ...AG. The upstream regions of introns are pyrimidine rich, the coding region of CYP1B1 gene starts at the 5' end of 2nd exon and ends within the last exon. The translation product of CYP1B1 gene is 543 amino acids (corresponding to 1,629 bases) long protein with 53 residue membrane bound N-terminal, 10 residue proline rich hinge (contributing to the flexibility of the protein) and 480 amino acid long cytosolic globular domain. The carboxy terminal region of CYP1B1 is highly conserved suggesting a pivotal role of this region. The carboxy terminal of cytochrome p450 enzymes corresponds to a set of conserved core structures (CCS) for the heme-binding region essential for the normal functioning of this class of enzymes. Figure 3 illustrates a schematic diagram of CYP1B1 gene. The CYP1B1 is a single copy gene as confirmed by the southern analysis studies using DNA probes to all three exons. It differs significantly from its close relatives CYP1A1 in CYP1A2. CYP1A1 and CYP1A2 both have seven exons as against three in CYP1B1 with former being located on chromosome 15 and the latter on chromosome 2. CYP1B1 gene lacks a consensus TATA box in the promoter region and contains nine TCDD responsive enhancer regions (5'-GCGTG-3') located within a 2.5 kB upstream. Deletion analysis studies with chloramphenicol acetyltransferase reporter gene constructs containing 5'CYP1B1 genomic fragment indicated that an upstream region from –1,002 to –835 contains core binding motifs contributing to the TCDD inducible expression. CYP1B1 is expressed in many tissues including brain and breast secretary cells^95^ and almost in 15 other nonocular tissues.^89^ In its metabolic pathway, CYP1B1 generates molecular species which operate through some signaling pathways and regulate expression of a spectrum of genes implicated in growth, development and differentiation of different ocular structures. CYP1B1 gene product also metabolizes vitamin A in two steps to all-transretinal (aldehyde form) and all-transretinoic acid (carboxylic acid form).^96^ All-transretinoic acid is an effective morphogen and regulates *in utero* (fetal) development, growth and differentiation.^97^ The CYP450 enzymes usually incorporate one atom of oxygen into its substrate creating hydroxyl (OH), amino (NH_2_) or carboxyl (COOH) functional groups (oxidoreductase functions), therefore, possibly playing a role in mediating oxygenation of bio-organic species and consequently signal transduction.^98,99^ CYP1B1 is expressed in ciliary body, iris, retina and also in TM of the anterior segment chamber of the eye.^100^ However, recently some investigators have put a caveat and observed that CYP1B1 is not expressed in TM at any stage of eye development^101^ suggesting that TM maintenance is being carried out by exotic supply of CYP1B1 protein to TM. This fact may provide a premise in designing a recombinant CYP1B1 product and delivering it for therapeutic use for TM dysgenesis.

Although, three different loci for PCG have been identified but the CYP1B1 gene located in GLC3A region (chromosomal location 2p21) is the first and was until recently the only gene to be implicated in etiology and pathogenesis of PCG.^102^ This gene was identified using positional cloning and *in situ* hybridization.^102^ CYP1B1 gene mutations are one of the major etiologies behind PCG. Mutations in this gene have gained a lot interest from researchers and now ocular geneticists are reporting increasing number of mutations. Till date more than 500 PCG patients with mutations in various regions of CYP1B1 gene have been reported all over the world^103^ (Ni La et al, 2011). These mutations occur in variable frequencies and pathogenicities. Some pathogenic mutations (e.g. Gly368Stop) are extensively widespread while others (e.g. p.Gly252Arg, p.Gly367Arg and p.Pro370Leu) occur in varying prevalence in different population. Mutations in CYP1B1 gene are considered to be the most common cause of PCG. Table 3 enlists a comprehensive update of all the mutations in CYP1B1 gene in PCG patients with the exact mutation and aberration in the translation product. The table also indicates the mutation type against each. Our research group has reported CYP1B1 mutations in 32 of total 73 PCG patients. We have identified and reported novel mutations (six nonsynonymous and one synonymous) p.Leu24Arg, p.Phe190Leu, p.Gly329Asp^104^ p.Ile94X, p.His279Asp, p.Gln340His and p.Lys433Lys^105^ along with other mutations in CYP1B1 gene in PCG patients. Table 4 enlists the novel mutations reported by our laboratory with their corresponding GenBank accession numbers and likely effect on the corresponding protein function. Our study constitutes the first CYPIB1 mutational spectrum report for PCG from northern India.^104^

**Fig. 4:**
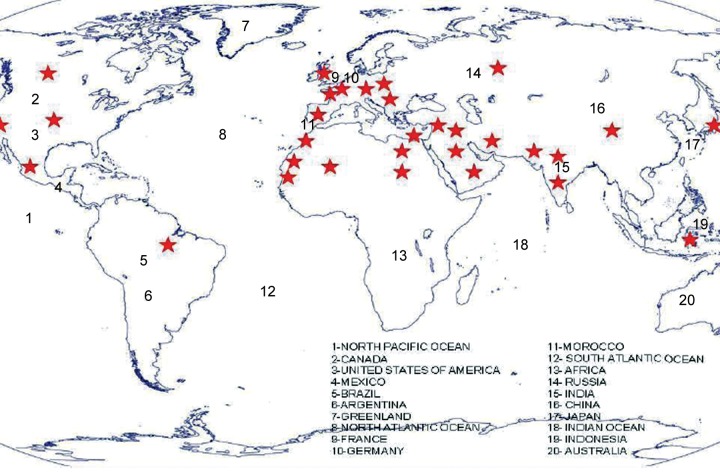
Worldwide distribution of CYP1B1 mutations (as reported in literature) in PCG. The stars show the areas where CYP1B1 mutation have been reported in PCG patients

Mutations in CYP1B1 gene have been described as the main underlying genetic etiology for majority of PCG cases in Turkish and Saudi Arabian families.^62^ European, Canadian and Slovakian families suffering from PCG have also been report to harbor mutations in CYP1B1 gene.^106^ Figure 4 illustrates the worldwide distribution of CYP1B1 mutations in PCG patients and a comprehensive country wise list has been shown in Table 5. A very large number of mutations in this gene have been reported with approximately 30% insertions and deletion.^34,62,102,107-113^ The number of reports of allelic heterogeneity at GL3CA locus is increasing very fast. Three-dimensional model structure analyses have revealed that most of the nonsynonymous mutations affect the relatively conserved carboxyl half of the CYP1B1 protein leading to derangements in the core structure of the enzyme.^114^ This, therefore, makes a good rationale to think that these mutations affect the functional properties of the enzyme like substrate recognition, etc. Our studies, for this reason, are currently focused on functional characterization of CYP1B1 mutations. The CYP1B1 polymorphism is most likely to be conserved; however, some modulations are possible within certain limits. In Saudi Arabia, the mutations G61E accounts for almost 70% of the PCG cases.^61,108,115^ Interestingly, E387K mutation was found in 43 patients who could be traced to a common ancestor.^115^ Stoilov et al reported 4340delG mutation in 20.2% of PCG cases in Brazil.^112^ Till date, at least 147 different CYP1B1 mutations have been identified all over the world in 542 PCG patients^103^ and the list is growing with increasing number of reports. These include deletions, insertions missense, nonsense, frameshift as well as truncating mutations and a mutation in noncoding region of exon I. Table 3 gives a comprehensive update on the exon-wise distribution of CYP1B1 mutations in PCG patients. Studies suggest that the most severe phenotype is associated with the frameshift mutations.^116^ The mutational spectrum of CYP1B1 gene in different population and its consequent correlation with etiology and pathogenesis of PCG has lead to the idea that CYP1B1 enzyme is important for development of the anterior chamber of eye. CYP1B1 protein has been found to be present in higher levels in fetal ocular structure as compared to adult eye, therefore, lending support to the idea. Since ciliary body secretes metalloproteases (an enzyme whose catalytic activity requires a metal ion), CYP1B1 having metabolic influence on these secretions can influence IOP.^117^ Additionally, CYP1B1 (–/–) mice (also designated as CYP1B1 null mice) have on the contrary of the belief been found to be nonglaucomatous with normal anterior chamber. But electron microscopy of the anterior chamber structure of such mice has revealed hypoplasia of the TM, abnormally placed basal lamina and iridocorneal adhesions.^118^ More recent reports have, on the other hand, reported elevated IOP in CYP1B1(–/–) mice.^119^ Also, CYP1B1 mutations in presence of tyrosinase (TYR) deficiency (129 x1/SvJ) develop into a more severe phenotype immediately suggesting TYR as a modifier gene.^118^ TYR deficiency is also found in patients with anterior segment dysgenesis (ASD) and albinism.^120^ Therefore, in addition to identifying other loci and genes as agents behind pathogenesis of PCG, gene-gene interaction studies might lead to proper understanding of the disease pathogenesis and consequently revealing probable mechanism for required therapy.

**Table 3:** **Table 3**: Mutations found till date in CYP1B1 gene in PCG patients. Only mutations that manifest in the translation product have been listed

*Genomic position*		*Base alteration*		*Change in protein*		*Mutation type*		*Number of cases reported*	
*Exon I*									
g.3131		C>T		Noncoding region		Probably regulatory		2	
*Exon II*									
g.3834		Insertion A		Frameshift		Insertion		11	
g.3860		C>T		p.Q19X		Nonsense		2	
g.3876		T>G		p.L24R*		Missense		2	
g.3905		Deletion 23bp		Deletion		Deletion in frame		2	
g.3913		C>T		p.Q37X		Nonsense		1	
g.3929		C>T		p.Q42X		Nonsense		1	
g.3956		Insertion C		Frameshift		Insertion		1	
g.3960		C>T		p.P52L		Missense		1	
g.3964		Deletion C		Frameshift		Deletion		1	
g.3972		Deletion C		Frameshift		Deletion		2	
g.3976		G>A		p.W57X		Nonsense		6	
g.3976		G>C		p.W57C		Missense		1	
g.3979		Deletion A		Frameshift		Deletion		1	
g.3985		C>G		p.I60M		Missense		1	
g.3987		G>A		p.G61E		Missense		207	
g.3988		Deletion A		Frameshift		Deletion		2	
g.4004		Deletion 8bp		Frameshift		Deletion		1	
g.4035		T>C		p.L77P		Missense		2	
g.4046		T>A		p.Y81N		Missense		2	
g.4048		C>A		p.Y81X		Nonsense		2	
g.4052		Deletion G		Frameshift*		Deletion		2	
g.4081		Deletion C		Frameshift		Deletion		2	
g.4089		T>C		p.V95A		Missense		1	
g.4122		C>A		p.A106D		Missense		1	
g.4124		C>G		p.L107V		Missense		4	
g.4133		C>T		p.Q110X		Nonsense		12	
g.4148		G>C		p.A115P		Missense		2	
g.4154		C>T		p.R117W		Missense		1	
g.4155		G>C		p.R117P		Missense		1	
g.4157		C>T		p.P118S		Missense		2	
g.4168		Insertion 18bp		Frameshift		Insertion		1	
g.4196		Deletion 5bp		Frameshift		Deletion		1	
g.4200		T>G		p.M132R		Missense		4	
g.4206		T>C		p.F134S		Missense		1	
g.4236		A>C		p.Q144P		Missense		1	
g.4236		A>G		p.Q144R		Missense		1	
g.4238		Deletion 10bp		Frameshift		Deletion		4	
g.4259/60		Deletion AT❶		Frameshift		Deletion		1	
g.4280		C>T		p.Q159X		Nonsense		1	
g.4292		C>T		p.R163C		Missense		1	
g.4306		Insertion T		Frameshift		Insertion		2	
g.4322		G>A		p.E173K		Missense		8	
g.4322		G>T		p.E173X		Nonsense		1	
g.4330/31		Deletion TG❶		Frameshift		Deletion		3	
g.4335		T>G		p.L177R		Missense		2	
g.4335		T>C		p.L177P		Missense		1	
g.4339		Deletion G		Frameshift		Deletion		24	
g.4340		Deletion G		Frameshift		Deletion		34	
g.4342		Deletion G		Frameshift		Deletion		1	
g.4373		T>C		p.F190L*		Missense		1	
g.4375		C>A		p.F190L		Missense		2	
g.4379		G>T		p.D192Y		Missense		1	
g.4380		A>T		p.D192V		Missense		4	
g.4383		C>T		p.P193L		Missense		4	
g.4397		G>A		p.V198L		Missense		2	
g.4410		C>A		p.A202D		Missense		1	
g.4413		A>G		p.N203S		Missense		1	
g.4430		T>C		p.C209R		Missense		1	
g.4449		G>T		p.S215I		Missense		3	
g.4490		G>A		p.E229K		Missense		29	
g.4499		G>C		p.G232R		Missense		1	
g.4520		A>C		p.S239R		Missense		4	
g.4523		Deletion C		Frameshift		Deletion		1	
g.4530		Duplication 16/		Frameshift		Duplication and deletion		1	
		Deletion 6❷							
g.4531		Deletion 22 bp		Frameshift		Deletion		1	
g.4547		C>T		p.Q248X		Nonsense		1	
g.4578		C>A		p.F261L		Missense		3	
g.4589		G>T		p.E262X		Nonsense		1	
g.4602		Deletion 9 bp		In frame deletion		Deletion		4	
g.4611		Duplication 9 bp		Frameshift		Duplication		2	
g.4633		Deletion C		Frameshift		Deletion		2	
g.4635		Deletion T		Frameshift		Deletion		5	
g.4640		C>G		p.H279D*		Missense		1	
g.4645		C>A		p.C280X		Nonsense		3	
g.4646		G>T		p.E280X		Nonsense		3	
g.4650		G>A		p.S282N		Missense		1	
g.4664		G>A		p.A287S		Missense		1	
g.4668		Insertion C		Frameshift		Insertion		6	
g.4673		Insertion C		Frameshift		Insertion		4	
g.4677		A>G		p.D291G		Missense		6	
g.4680/81❸		T>A/G>A		p.M292K		Missense		1	
g.4761		A>G		p.N319S		Missense		1	
g.4763		G>T		p.V32L		Missense		2	
g.4776		Insertion AT		Frameshift		Insertion		4	
g.4791		G>T		p.G329V		Missense		5	
g.4791		G>A		p.G329D*		Missense		2	
g.4793/94		G>T/C>T		p.A330F		Missense		1	
g.4812		C>A		p.S336Y		Missense		1	
g.4825		G>T		p.Q340H*		Missense		1	
g.4828		G>A		p.W341X		Nonsense		1	
g.4838		Deletion CTC		In frame deletion		Deletion		1	
*Intron-II-Exon-III junction*									
g.4849		Deletion Intron-II-Exon-III		Frameshift		Deletion		2	
*Exon-III*									
g.7899		Deletion 12bp		In frame deletion		Deletion		1	
g.7900		C>T		p.R355X		Nonsense		2	
g.7900		Deletion CG		Frameshift		Deletion		4	
g.7901		Deletion 13bp		Frameshift		Deletion		30	
g.7925		T>A		p.V363D		Missense		2	
g.7927		G>A		p.V364M		Alternate frame		17	
g.7930		G>T		p.G365W		Missense		2	
g.7934		Deletion G		Frameshift		Deletion		2	
g.7939		C>T		p.R368C		Missense		3	
g.7940		G>A		p.R368H		Missense		87	
g.7940		G>T		p.R368L		Missense		2	
g.7945		Deletion C		Frameshift		Deletion		2	
g.7957		G>A		p.D374N		Missense		8	
g.7959		C>G		p.D374E		Missense		2	
g.7970		T>A		p.L378Q		Missense		1	
g.7990		C>T		p.L385F		Missense		14	
g.7996		G>A		p.E387K		Missense		65	
g.7999		G>A		p.A388T		Missense		3	
g.8005		C>T		p.R390C		Missense		23	
g.8005		C>A		p.R390S		Missense		4	
g.8006		G>A		p.R390H		Missense		74	
g.8033		T>G		p.I399S		Missense		1	
g.8034		C>T		p.P400S		Missense		3	
g.8037		Duplication 10bp		Frameshift		Duplication		39	
g.8047		Duplication 10bp		Frameshift		Duplication		1	
g.8104		A>T		p.N423Y		Missense		1	
g.8111		Insertion G		Frameshift		Insertion		2	
g.8127		C>G		p.D430E		Missense		1	
g.8131		C>G		p.L432V		Missense		1	
g.8139		G>A		p.W434X		Nonsense		1	
g.8147		C>T		p.P437L		Missense		7	
g.8162		C>G		p.P442R		Missense		1	
g.8165		C>G		p.A443G		Missense		3	
g.8167		C>T		p.R444X		Nonsense		1	
g.8168		G>A		p.R444Q		Missense		9	
g.8170		T>A		p.F445I		Missense		2	
g.8171		T>G		p.F445C		Missense		1	
g.8171		T>C		p.F445S		Missense		2	
g.8182		Deletion G		Frameshift		Deletion		11	
g.8209		Deletion '		Frameshift		Deletion/insertion		1	
		5bp/insertion							
		11bp❹							
g.8214		Duplication 27bp		Frameshift		Duplication		3	
g.8214/15		Deletion AG		Frameshift		Deletion		2	
g.8234		G>A		p.G466D		Missense		2	
g.8240		Duplication 27bp		Frameshift		Duplication		2	
g.8242		C>T		p.R469W		Missense		53	
g.8246		G>A		p.C470Y		Missense		2	
g.8249		T>G		p.I471S		Missense		2	
g.8297		T>C		p.L487P		Missense		2	
g.8329		A>G		p.N498D		Missense		1	
g.8333		A>G		p.E499G		Missense		1	
g.8341		Deletion A		Frameshift		Deletion		2	
g.8354		Deletion 20bp		Frameshift		Deletion		2	
g.8373		Deletion 6bp		In frame deletion		Deletion		2	
g.8405		G>A		p.R523K		Missense		4	
*Undefined*									
Not defined		Not defined		Not defined		Not defined		89	
Total number of mutation types				147 (excluding unidentified types)		Total number of patients		542	

*These mutations were first reported from our laboratory;

❶An example of double deletion; ❷Duplication and deletion in the same gene; ❸Double substitution; ❹Deletion and insertion in the same gene

**Table 4:** **Table 4:** Novel mutation in CYP1B1 gene in PCG patients that we have reported

*Mutation*		*Codon change*		*Amino acid change*		*Mutation type*		*GenBank acc. number*		*Likely effect*	
g. 38159965 T>G		CTG to CGG		p.Leu24Arg		Nonsynonymous		FJ815437		Pathogenic	
g. 38155466 C>A		TTC to TTA		p.Phe190Leu		Nonsynonymous		FJ815438		Pathogenic	
g. 38155050 G>A		GGC to GAC		p.Gly329Asp		Nonsynonymous		FJ815439		Pathogenic	
g. 38302285 Gdel		insSTOP		p.Ile94X		Nonsense		GQ925803		Pathogenic	
g. 38301697 C>G		CAC to GAC		His279Asp		Nonsynonymous		GQ925804		Pathogenic	
g. 38301512 G>T		CAG to CAT		p.Gln340His		Nonsynonymous		GQ925805		Probably pathogenic	
g. 38298198 G>A		AAG to AAA		p.Lys433Lys		Synonymous		GQ925806		Nonpathogenic	

### Myocilin/TIGR Gene (Myocilin/Trabecular Meshwork Inducible Glucocorticoid Response Protein) and PCG

**Fig. 5:**
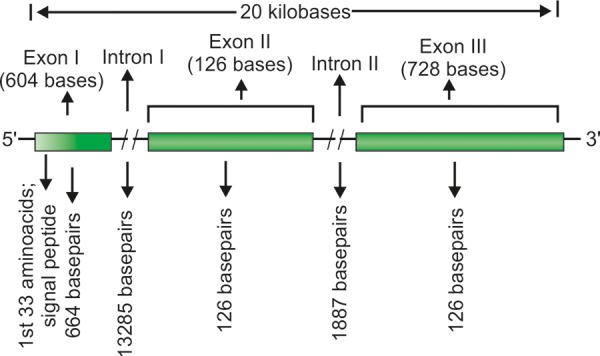
Schematic representation of MYOC gene

MYOC gene belongs to the family of the soluble N-ethylmaleimide sensitive factor attachment protein receptor (SNARE). The proteins of SNARE family function as vesicle trafficking and targeting factors thereby serving as molecular addresses on secretory vesicles.^121^ The MYOC gene codes for a protein that was initially named trabecular meshwork inducible glucocorticoid response protein (TIGR). This gene has been mapped to chromosomal locus 1q23-24^122^ with physical map between four contiguous genes *viz* SELL, SELE, APT1LG1 and AT3.^123^ Owing to its similarities with bullfrog olfactomedin and *Dictyostelium discoideum* myosin as per the sequence analogy, this protein was named myocilin by Kubota et al.^124^ MYOC spans an approximate of 20 kB and like CYP1B1 has three exons^116-118^ with exon I comprising of 604 bases, exon II 126 and exon III 728 bases. A schematic representation of the MYOC gene is given in Figure 5. There are presumably a good number of transcription regulatory sequences identified in the upstream region of the MYOC gene. Three different polyadenylation sites have been identified to be positioned in the 3' untranslated region (UTR) of the gene occupying positions 1,714, 1,864 and 2,006 downstream from the putative start codon. Its anticipated open reading frame has two possible start (ATG) sites adjoining each other. The translation product of MYOC gene is a 55 kDa olfactomedin-related secretory protein 504 amino acids long.^125^ In humans, the first 33 amino acids of this protein form a signal peptide and amino acids from 111 to 184 structure an alpha helical coiled region approximating the shape of myosin tail containing a leucine zipper motif involved in myocilin-myocilin interactions. Examination for hydrophobicity has interestingly revealed a hydrophobic region between amino acid 17 and 37, 426 and 244. The three amino acids at the carboxy terminal of human MYOC protein are serine, lysine and methionine which is a peroxisome targeting sequence in other proteins.^126^ MYOC protein is expressed in many ocular tissues like sclera, ciliary body, retina, TM, etc. It is also expressed in a variety of nonocular tissues like myocardium, lungs, pancreas, etc.^124^ Despite exhaustive research, no function as of now has been attributed to this protein though it has been functionally reported to be the TIGR. The expression pattern of normal and mutant MYOC in cultured ocular and nonocular cells has been studied by Jacobson group^127^ with findings that normal MYOC is secreted from the cultured cells but very little or none from cells expressing five different mutant forms of MYOC. This suggests that glaucoma precipitates either due to inadequate levels of secreted MYOC or hampered TM cell function. This may presumably be caused by obstructions in the TM secretory pathway. Mature MYOC forms aggregates (multimers) secreted into trabecular extracellular matrix (ECM) interacting with various ECM components. Although mutations in CYP1B1 and myocilin (MYOC) genes have been implicated in PCG and POAG respectively, variants in both the genes have been observed in both PCG and POAG. These observations indicate an intricate and multifaceted genetics involved in PCG. In case of POAG, mutations in at least three genes, *viz* MYOC^102^ optineurin (OPTN)^62^ and WDR36^109^, have been implicated accounting for 3 to 4% of total number of cases. In a recent study, it was found that mutations in CYP1B1 alone can be underlying cause in POAG.^108^ In addition, there is growing body of evidence suggesting that there exists some functional interaction between CYP1B1 and MYOC^34^ and this fact further lends support to the observation that PCG can also be caused by mutations in MYOC (MYOC playing the role of a potential modifier gene).^107^ This digenic inheritance engrossing both MYOC and CYP1B1 immediately indicates the role of MYOC in PCG (PCG in this case being allelic variant of POAG). MYOC gene, initially found to be associated with POAG was the first gene found to be implicated with any type of glaucoma. MYOC was identified in a study correlating effects of dexamethasone on cultures of TM cells.^100^ This gene, being the first to be associated with glaucoma, was named GLC1A^111,112^ in accordance with HGO genome database nomenclature. Like CYP1B1, this gene also is expressed in many tissues of the body including the ocular ones^113,114^ with highest concentration in the iris, sclera and TM.^108,112^ It has been observed that individuals with mutations in MYOC tend to have higher IOPs implying its role in PCG.^110,119,120^ We have already reported five single nucleotide polymorphisms (SNPs) *viz* –126T > C, –83G > A, p.R76K, IVS2 + 35G > A and p.Y347Y in MYOC gene analysis in our studies on gene analysis of PCG patients.^128^

**Table 5:** **Table 5:** Geography/ethnicity-based distribution of mutations in CYP1B1 gene in PCG patients

*Country/ethnogeographic origin*		*Number of cases reported*		*References*	
Algeria		15		113❶	
Asia		4		148❷	
Brazil		26		53	
		4		149	
		9		150	
Britain		15		151❸	
Canadian		2		152	
China		7		153	
		1		154	
		20		155	
		6		156	
		1		157	
Ecuador		2		158	
Germany		9		159	
Gypsies		7		160	
Hispanic		17		106❹	
India		37		161	
		2		162	
		24		163	
		1		116	
		1		164	
		1		165	
		6		166	
		23		104	
		9		105	
Indonesia		6		167❺	
Iran		72		168	
		13		169	
Israel		9		170	
Japan		13		110	
		2		107	
		4		171	
Kuwait		12		172	
Mexico		4		173	
		2		174	
Morocco		11		175	
		19		176	
Netherlands		1		177	
		1		178	
Oman		8		179	
Pakistan		3		180	
Russia		26		181❻	
Saudi Arabia		24		62	
		10		108	
		5		182❼	
Slovak Gypsies		20		115	
Spain		14		183	
		1		184	
Turkey		5		102	
		1		185	
		15		186	
United States of America		1		187	

❶This study also included French and Portuguese patients; ❷This study also included Hispanic, Middle Eastern and Caucasian patients; ❸This study also included Italian and Indian patients; ❹This study also included American, French, British and Turkish patients; ❺This study also included Sudanese, Turkish and Italian patients; ❻This study also included Costa Rican, Turkish, German, Swiss, American and Saudi Arabian patients; ❼This study also included Egyptian patients

### Forkhead-related Transcription Factor C1

**Fig. 6:**
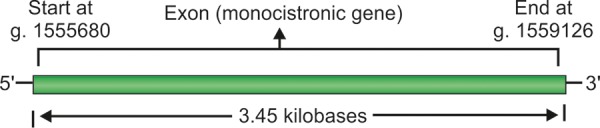
Schematic representation of FOXC1 gene

FOXC1 is another potential etiological factor implicated in PCG pathogenesis. It is Forkhead-related transcription factor C1 (FOXC1 or FKHL7) and is located on p-arm of chromosome 6 (locus 6p25). See Figure 6 for a schematic representation of FOXC1 gene. FOXC1 mutations in PCG recently got attention. FOXC1 mutations were previously known to be directly involved in eye conditions collectively referred to as ASD. As TM (tissue deranged in PCG) is the part of the anterior segment, it is logical to think of its role in PCG development and pathogenesis. In our studies, we found two sequence variations *viz* GGC375ins and GGC447ins in FOXC1 gene in PCG patients who did not harbor mutations in CYP1B1 gene. However, it is worth mentioning that no significant correlation could be observed between FOXC1 gene mutations and PCG in our patient cohort.^128^

### Latent Transforming Growth Factor Beta Binding Protein 2

**Fig. 7:**
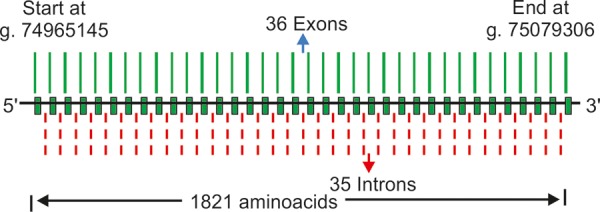
Schematic representation of LTBP2 gene

Linkage analysis studies of PCG in consanguineous Pakistani PCG families were recently reported and showed involvement of a new chromosomal locus adjacent to GLC3C on 14q24.2-24.3.^3^ The candidate gene identified was latent transforming growth factors-binding protein 2 (LTBP2). Figure 7 depicts a schematic diagram of this gene. Ali et al^129^ reported truncating mutation in this gene in PCG patients.^129,130^ The expression network of LTBP2 in the TM, ciliary bodies and ciliary processes^129^ has augmented to the complexity in the mechanism of PCG. In our studies, we also screened 54 PCG patients (who were negative for mutations in CYP1B1, MYOC and FOXC1) for mutations in LTBP2 and compared them with 50 controls. We found one intronic SNP (rs3742793) between exon VI and exon VII in 18 patients. Our studies suggest no role of mutations in LTBP2 in PCG^131^

### Other Genes

Other genes thought to play a role in the pathogenesis of PCG are optineurin (OPTN), WDR36, LOXL1, PAX6, PITX2, etc. but no conclusive report to confirm their role in PCG etiology has come about. However, it is sure that the more we advance in the studies of PCG; we are likely to identify many more genes having role in the etiology of this disorder as well as other related disorders termed as ASD.^14^

### Role of Mitochondria

Mitochondria (singular: mitochondrion) are double membrane bound, roughly sausage shaped, maternally inherited organelles which play role in energy conversions and many other processes in the cell. They are present in all cells of the body except red blood corpuscles (RBCs). The mitochondria are semiautonomous organelles containing their own genome which is circular, double-stranded, nonhistone bound DNA coding for a variety of enzymes important in oxidative phosphorylation (OXPHOS). The human mitochondrial DNA (mt-DNA) contains 16,569 base pairs comprising 37 genes which regulate oxidative phosphorylation. Out of these 37 genes, 13 code for different subunits of the respiratory chain enzymes, 22 for different transfer RNAs (tRNAs) and the remaining 2 for ribosomal RNAs (rRNAs). The genes in mt-DNA lack introns and, therefore, do not have any intervening sequences. This is very important to note that since mitochondria are the site for electron transport chain (ETC) and their DNA is naked; it follows that mt-DNA is more prone to mutations as compared to genomic DNA. And since mitochondria are maternally inherited, vertical transmission through them is inevitable. Having primitive DNA repair mechanism^132^ makes mt-DNA further vulnerable to mutations often leading to cell death^133^ through elevation in ROS and apoptosis. A large number of human diseases have been associated with mutations in mt-DNA which range from muscular dystrophy to infertility^134^ but those involving ocular structures are Leber's hereditary optic neuropathy (LHON),^135^ pseudoexfoliation glaucoma (PEG), POAG, PACG and PCG.

There is a very high concentration of mitochondria present in the cells of optic nerve head presumably because of the requirement of higher amount of energy as compared to other tissues. Hence, the very survival of the RGCs of the optic nerve head depends on its mitochondria^137^ Being involved in apoptosis, calcium signaling and metabolism of reactive oxygen species (ROS); mitochondria form the imperative organelles for the survival of RGCs. It is well known that mitochondrial dysfunction/malfunction leads to increase in oxidative stress (OS) consequently ensuing damage to ETC enzymes, genomic DNA, mt-DNA and impairment in regulating calcium. This gamut of derangements results in neuronal degeneration^138^ finally precipitating glaucoma and other related conditions. The observation that glaucoma is accompanied by increase in OS and decrease in antioxidant activity appends further support to this idea.^139^ OS elevation leads to injury in the anterior segment structures of the eye.^140^ Furthermore, it has been observed that oxidative damage to TM is increased in glaucomatous patients^136^ causing increased IOP (one of the main etiological factors for PCG). Up until now, numerous studies have been published which suggest mitochondrial dysfunction as the etiological factors for various glaucomas.^138,141,142^ All these factors may be involved directly or indirectly in PCG. Abu Amero et al^136^ and Izzoti et al^143^ reported that mutations in mt-DNA are present in cases of PACG. Further, an increased frequency of mutations in mt-DNA has been reported in POAG, PACG as well PEG cases.^144,145^ With regards to PCG, we compared 35 PCG patients with 40 controls and screened them for mutations in mitochondrial genome by analyzing the sequences against reference sequence NC-012920. We found that 22.85% PCG patients had potentially pathogenic sequence changes in mt-DNA (as per the PolyPhen and SIFT analysis) and 57.14% had sequence changes associated with increase in ROS.^146^ In these studies, the mitochondrial sequence variations in PCG patients were very high as compared to the controls, therefore, establishing mitochondria as the hot spot for etiology of PCG. In our other studies, we found the similar results.^147^ We have also proposed the probable mechanism for mitochondrial mutation induced TM dysgenesis.^146^

## CONCLUSION

PCG is a complex neurodegenerative disorder with established genetic etiology. It is mainly inherited through autosomal recessive mode of inheritance. The number of chromosomal aberrations, many chromosomal loci identified and a great variety of genes involved make PCG an intricate eye condition with multifarious etiologies and consequently diverse pathogenic mechanisms. This diversity in pathogenesis accounts for the variable penetrance observed in PCG. Though a good number of studies have been published but many more rigorously investigative strategies need to be developed in order to get further insight into this blinding disorder. Many studies about the mutational spectrum of PCG in different population are being continuously reported. This will aid in correlating pathogenic mutations with the disease phenotype. The more we know about the pathogenesis of this disease, the more effectively its management strategem can be planned so that patients and their relatives are counseled with respect to risk factors. This may improve the outcome of the management regimens. Molecular evaluation of PCG is necessary and screening of high risk group can lead to good outcomes. With regards to mitochondrial mutations and increased OS, prompt antioxidant therapy and genetic counseling will and should form an essential part of glaucoma therapy in near future though gene therapy regimen is yet far from being a reality. In near future, recombinant CYP1B1 proteins might find a therapeutic role in PCG management if the CYP1B1 mutation in a patient is identified. Moreover, stem cell therapy with the correct copy of the implicated genes may be a promising regimen if the molecular mechanism of PCG is worked out in detail. Additionally, understanding the etiology, pathogenesis, mechanism and mode of PCG inheritance can help in devising counseling strategies to keep family members and children of affected members at bay. Additionally, marriage counseling can also be practiced to bring down the prevalence of PCG particularly in consanguineous societies. It is, therefore, endorsed that every ophthalmology hospital should have a separate unit for genetic evaluation, molecular workout and genetic counseling of patients suffering from PCG and other related ocular diseases.
